# Adverse drug reaction reporting and monitoring: A review of global practices

**DOI:** 10.6026/973206300214435

**Published:** 2025-12-15

**Authors:** Nehal Mohsin, Ali Mohammed Alyami, Ibrahim Mohammed Al Shermah, Ali Mohammed Al Shermah, Khalid Mohammed Alharthi, Turki Morshed Alyami, Hussain Abbas Almakrami, Hassan Hussain Mohammed Muqmish, Ibrahim Jaber Ali Sarrar

**Affiliations:** 1 Department of Pharmacology, College of Pharmacy, Najran University, Najran, Saudi Arabia

**Keywords:** Adverse drug reactions, pharmacovigilance, drug safety monitoring, spontaneous reporting, regulatory systems, patient safety, global health

## Abstract

The negative drug reactions (ADRs) are a major challenge to the global health due to their contribution to morbidity and mortality as well as the increased healthcare expenditure. Therefore, it is of interest to explore the international contextual situation of ADR reporting and monitoring systems, their development, their presence in the market, and their future perspectives. The review provides an overview of different pharmacovigilance frameworks put in place by international organizations and national regulatory bodies and their weaknesses and strengths. It was found that despite the tremendous progress achieved in the formation of global pharmacovigilance networks, much still needs to be done in the detection, reporting, and prevention of ADRs especially in the low and middle-income countries. The review identifies new trends and possible innovations that can change ADR monitoring in the decade and provide patient- centered reporting, integrate real-world evidence, and predictive toxicology methods.

## Background:

Adverse drug reactions (ADRs) are among the most important issues in the contemporary healthcare system that lead to a considerable morbidity and mortality of patients in addition to consuming a significant part of the healthcare costs of the population across the globe. Research estimates that ADRs dominate about 5-10 percent of all hospital admissions with even greater rates being reported in the aged groups [1]. According to the definition provided by the World Health Organization (WHO), ADR is considered as a reaction to a drug that is noxious, accidental, and happens at doses typically utilized in man to prevent disease, diagnose it, and/or to treat it, or to alter physiologic activity [2]. Although ADRs are often identified after drugs adopt extensive clinical use, rigorous pre-marketing clinical trials have not enabled the detection of many ADRs. Pharmacovigilance, the science and practices of detecting, evaluating, comprehending and averting drug side-effects or any other type of drug-related issue, has progressed greatly because the thalidomide disaster of the 1960s brought to attention the necessity of conducting extensive surveillance of the safety of drugs [3]. Currently, the system of ADR monitoring has been established in virtually every country, although it is more effective, more comprehensive, and more implemented in various regions in different ways. According to current information, despite decades of work on the development of pharmacovigilance, underreporting is a widespread problem in all countries of the world, and it is estimated that only 5-10% of all ADRs are reported to the regulatory authorities [4]. This is also referred to as the iceberg problem, which is one of the major obstacles to the full evaluation of drug safety. Also, there are still controversies about the best reporting methods, the ratio between spontaneous and active surveillance, and how the emerging digital technologies can be implemented into the existing pharmacovigilance systems. The reason why this review is important is due to the growing globalization of pharmaceutical markets, the accelerating launch of complex biologic therapies, and the acknowledgement of ADRs as an important issue of patient safety. With a growing pressure on healthcare systems all over the world to guarantee medication safety and control expenses, it is becoming important to learn more about the global environment of ADR reporting and monitoring. Therefore, it is of interest to (1) review the history and the modern status of pharmacovigilance systems in different parts of the world; (2) discuss the various methods of ADR monitoring in different regions and health care settings; (3) pinpoint the deficiencies in the history and current solutions in ADR detection and reporting; and (4) outline the future prospects that may help improve the drug safety monitoring systems in various locations.

## Historical development of pharmacovigilance:

Modern pharmacovigilance started to develop on the basis of numerous drug safety catastrophes in the course of the 20th century. The development of systematic drug safety surveillance programs took place as an outcome of the thalidomide tragedy of the late 1950s and early 1960s, which caused thousands of birth defects all over the world [5]. The WHO has responded to this crisis by setting up the Programme for International Drug Monitoring (PIDM) in 1968, which was a collaboration framework that allowed countries to exchange ADR data [6]. This program would be the start of formal cooperation in pharmacovigilance at international level and it has expanded to more than 150 member countries. During the 1970s and 1980s, most of the high-income nations developed national pharmacovigilance centers that had different levels of regulatory powers. In 1993, the United States introduced the MedWatch program, the single system of healthcare professionals and consumers reporting severe adverse events [7]. The European Union established its pharmacovigilance system in the form of a progression of directives, the final of which was the pharmacovigilance legislation 2010 which harmonized the reporting requirements among the member states [8]. The idea of spontaneous reporting systems (SRS) assumed the center stage of the pharmacovigilance actions at this time. These systems are based on voluntary reporting of suspected ADRs by the health care professionals, patients and manufacturers. In 1978, the WHO became the largest database of single case safety reports in the world, with more than 25 million reports to date in VigiBase [9]. This repository in the entire world allows detecting signals by processing the aggregation of information of several countries.

The 20th and early 21st centuries saw a great deal of methodological development in pharmacovigilance. Some statistical techniques, including disproportionality analysis (e.g., Reporting Odds Ratio, Proportional Reporting Ratio) were devised to reveal possible safety indicators with big SRS databases [10]. These are useful in terms of their role in hypothesis generation but they have limitations such as the fact that they cannot be used to determine causality or even incidences. Technological advances have also influenced the formation of pharmacovigilance. Electronic reporting systems have enhanced efficiency and the quality of data in most environments because of the change that occurred between paper-based and electronic reporting systems. More recently, automated signal detection and analysis has become accessible to the combination of artificial intelligence and machine learning approaches [11]. After these developments, the old problem of underreporting remains, so it is worth investigating the addition of other methods, including active surveillance and generation of real-world evidence.

## Global pharmacovigilance frameworks:

The international pharmacovigilance environment has been described as a sophisticated web of international, regional and national systems that are in place to facilitate safety of drugs across the borders. PIDM of WHO is the focal point of the international pharmacovigilance activities and offers technical assistance to member countries by maintaining the VigiBase as the global database of individual case safety reports [12]. This system has a scientific and operational centre in Uppsala, Sweden, at the WHO Collaborating Centre of International Drug Monitoring that formulates methods and standards of ADR assessment. On the regional level, a number of harmonization efforts have been developed in order to cope with the issues of global pharmaceutical markets. Guidelines on pharmacovigilance have been developed by the International Council of Harmonisation of Technical Requirements in Pharmaceuticals to Human Use (ICH), comprising of E2 series on pharmacovigilance planning and the MedDRA (Medical Dictionary to Regulatory Activities) coding adverse events terminology [13]. The standards enable the uniformity of data acquisition and sharing between various regulatory systems. EMA has developed a very elaborate system of pharmacovigilance regionally with its EudraVigilance database through which all reports of suspected adverse reactions in the European Economic Area are processed [14]. The 2012 pharmacovigilance legislation brought about some innovations such as patient reporting, periodic safety update reports and further monitoring of some medicinal products. This system proves to be advantageous in terms of regional integration and at the same time the national pharmacovigilance centers serve as the main points of contact. The FDA Adverse Event Reporting System (FAERS) is a database that stores data on adverse event and medication error reports reported to the FDA in North America by the US Food and Drug Administration (FDA) [15]. The Sentinel System of the FDA, which was introduced in 2008, is one of the steps towards active surveillance based on the use of big electronic healthcare databases to assess the safety of medical products [16]. There are certain limitations of the spontaneous reporting method that are addressed with this complementary method: the latter allows conducting more systematic evaluation of drug safety in specific populations. The Asian-Pacific pharmacovigilance situation is rather heterogeneous, and healthcare system and regulatory abilities are different. In Japan, the Pharmaceuticals and Medical Devices Agency (PMDA) operates a pharmacovigilance system that combines spontaneous adverse drug reaction reporting with structured post-marketing surveillance measures, including postmarketing all-case surveillance and other observational studies [17]. National Center of ADR Monitoring in China has developed on a booming basis in recent years but still there are some difficulties in completeness of reporting and signal detection [18]. The ASEAN countries have also developed a harmonized network of pharmacovigilance to promote sharing of information and capacity building in the region. Practical problems encountered by low- and middle-income countries (LMICs) in developing an efficient pharmacovigilance system include deficiency of resources and infrastructure, and conflicting health priorities. The Global Pharmacovigilance Programme of the WHO has put in place a number of programs to assist these nations which include instituting regional pharmacovigilance centers, training programs and so on [19]. However, with such endeavors, a good number of LMICs still face low reporting rates and low signal detection and assessment capability.

## National ADR reporting systems: 

## A comparative analysis:

The national ADR reporting systems can differ significantly in their constructions, coverage, and efficiency depending on the country. These differences are due to the differences in healthcare systems, regulatory measures, availability of resources, and cultural disparity in drug safety. A comparative analysis of these systems allows seeing some key insights into good practice and areas of ongoing concern in pharmacovigilance. In wealthy nations, the national pharmacovigilance centers are usually established in the full-fledged regulatory framework with special funding and personnel. Started in 1964, the Yellow Card Scheme of the United Kingdom has been one of the oldest and most comprehensive systems of national reporting [20]. It has now developed reporting by professionals and patients and electronic submission and mobile applications have helped to increase its ease of access. The Clinical Practice Research Datalink integration into the system permits the more effective identification of the signal, because spontaneous reports are observed together with the prescription data. The MedWatch program of the United States is a program that has a complex regulatory framework whereby it reports directly to the FDA and by the manufacturers [21]. Such a two-way approach poses a problem of standardization and completeness of data, but ensures a high level of coverage of the pharmaceutical market. The Safe Use Initiative by the FDA is a new effort to minimize the harm caused by the use of medications that is preventable, it is an addition to the existing ADR reporting programs with specific risk reduction measures. National pharmacovigilance in Scandinavian countries has the advantage of excellent healthcare databases facilitating both active and passive surveillance as well as the spontaneous reporting. The Danish National Prescription Registry of Denmark, which is connected with the hospital discharge diagnoses, offers an effective tool to research the drug safety in the real environment [22]. These integrated systems show the importance of integrating more data sources to obtain more comprehensive pharmacovigilance.

The challenges emerging economies are confronted with, in terms of creating an effective system of national reporting, are different. Started in 2010, the Pharmacovigilance Programme of India (PvPI) in India has grown substantially but still faces the issue of underreporting and its regional unequal distribution [23]. The effectiveness of the program in setting up peripheral pharmacovigilance centers in the country is a good example that can be emulated by other large and diversified countries in their efforts to enhance drug safety surveillance. The National Pharmacovigilance System of Brazil has already introduced some original methods of involving healthcare professionals, such as the creation of a mobile app that provides ADR reporting [24]. These online technologies help solve the geographical problem of a big nation with unequal access to healthcare. The integration of the system with the hospital information systems in large academic centers is a step towards a more automated ADR detection.

The pharmacovigilance infrastructure in African countries is usually less developed, although a number of countries have achieved much progress in recent years. The Pharmacovigilance Programme of South Africa enjoys a fairly high regulatory capacity, and academic support [25]. The African Pharmacovigilance Network was established in 2013 and has enabled the collaboration and capacity building at the regional level, but the lack of resources is a major challenge in the continent. Analysis of the national systems comparison has shown that a number of significant factors are related to effective pharmacovigilance: a specific funding source and regulatory agency; integration into the healthcare information system; the involvement of healthcare professionals and patients; and involvement in the international collaboration. These components offer a guideline of reinforcing the national ADR reporting systems in the world.

## Digital technologies in ADR monitoring:

Digitization of healthcare has brought out new opportunities and challenges to ADR monitoring. The detection, reporting and analysis of adverse drug reactions is transforming with the use of electronic health records (EHRs), mobile health applications, social media platforms, and artificial intelligence technologies. These online strategies have the opportunities to address the issues of long-term pharmacovigilance especially the challenge of underreporting. EHRs are an encouraging avenue of automated ADR identification using natural language processing and structured data processing. It has been shown that algorithms can find possible ADRs on clinical narratives with a reasonable amount of accuracy, but that it is difficult to separate actual drug-induced events and disease progression [26]. Sentinel System by FDA has led the way in implementing distributed data networks in a series of healthcare organizations as an active method to perform drug safety signal surveillance [27]. This method allows faster evaluation of the possible safety concerns and safeguards patient privacy by means of distributed data analysis. ADR reporting mobile applications have become widespread over the last few years and provided easy-to-use reporting solutions to healthcare professionals and patients. Examples of national pharmacovigilance programs that have adopted mobile technology include the Yellow Card app in the UK, MPA app in Sweden and PvPI app in India [28]. These applications should be able to enhance the rate and timeliness of reporting, but the effect on the overall signal detection has yet to be determined. These apps can also have barcode scanning and medication databases, which could enhance the completeness and accuracy of reporting. Social media tools have become unorthodox yet possibly useful to obtain pharmacovigilance data. On social networking sites, like Twitter, Facebook, and special health forums, patients can share their medication experiences in practice, and through this they can offer real-life information about the effect of drugs that may otherwise go unreported [29]. Multiple research works illustrated that it is possible to apply natural language processing to determine possible ADRs based on a social media publication, but issues of data quality, representativeness, and validation have been observed. The My Studies platform of the FDA can be seen as a step to the systematic gathering of patient-reported outcomes, such as adverse events, on a digital platform. Signal detection and assessment in the pharmacovigilance can be reshaped due to artificial intelligence and machine learning. Deep learning algorithms have the ability to find intricate patterns in massive datasets which could not be found through standard statistical techniques [30]. These methods have potential to identify rare or unexpected ADRs, which risk factors can lead to adverse events, and which patients might be at risk of developing certain drug toxicities. Combining genomics data with pharmacovigilance data is one of the new frontiers of individual drug safety evaluation. The blockchain technology has potential resolutions to the issues of data integrity and traceability in the field of pharmacovigilance. The immutability and decentralized quality of blockchain documentation may improve the privacy and openness of the ADR reporting along with the necessary privacy levels [31]. Pilot projects have investigated blockchain experts to trace drug distribution chains and confirm medication authenticity and they may be applied in pharmacovigilance. Regardless of these technological innovations, there are still quite serious issues regarding the implementation of digital solutions in pharmacovigilance. The problems of data standardization, interoperability, privacy protection, and algorithm bias should be resolved to achieve the maximum potential of these technologies. Moreover, the digital divide can potentially enhance the pre-existing disparities in pharmacovigilance capacity between the high-income and LMICs, and hence increase disparities in drug safety surveillance across the globe.

## Challenges in ADR Reporting:

Although the pharmacovigilance development has been decades, there are still serious issues in ADR reporting and monitoring across the globe. These obstacles are of methodological, practical and systemic nature and cannot be resolved without multidimensional solutions. These barriers should be known to enhance the safety of drug monitoring and prevent unnecessary harm to patients. The issue of underreporting is the most widespread in pharmacovigilance in all countries and healthcare systems. Research also shows that very few real ADRs are reported to the regulatory bodies, and estimates on this vary between 5-10 percent in high-income countries to even lower rates in resource-constrained environments [32]. ADR reporting has some serious blind spots due to the so-called iceberg phenomenon of such reporting, which may even postpone the identification of critical safety signals. There are various causes of underreporting such as time constraints and the absence of knowledge on causal factors and fear of prosecution, and the ignorance of healthcare professionals towards reporting procedures. Quality of ADR reports is another major challenge. Lack of complete information on the characteristics of patients, drug exposure, timing of events, and outcomes reduces the utility of most of the reports in detecting and assessing signals. National reporting systems have been studied and were found to vary significantly in the completeness of the reporting, with essential information left out in a significant percentage [33]. The use of standardized reporting forms and electronic systems including mandatory fields has enhanced the quality of data in certain contexts, yet there are still difficulties in making sure that clinical relevance and accuracy of information reported is accurate. The methodological problem in pharmacovigilance is causality assessment. Many structured algorithms for ADR causality assessment, such as the WHO-Uppsala Monitoring Centre (WHO-UMC) system and the Naranjo algorithm, have been developed to standardize decisions, but there is still no universally accepted gold standard and inter-rater agreement remains imperfect [34].

Pharmacovigilance systems find it especially difficult to detect uncommon and long-term ADRs. The spontaneous reporting systems are quite effective in the detection of the common, acute adverse reactions, but not very sensitive to rare occurrences or those that have long latency periods. Active surveillance methods that involve big healthcare databases can overcome some of these limitations but they need advanced methodology to adjust the confounding variables [35]. Increasing complexity of biologic drugs and combination therapies adds additional complexity to the measurement of infrequent adverse events. ADR monitoring poses special problems with special populations. There are various adverse events in pediatric patients that are not observed in adults and the safety of the drug is also dependent on the developmental factors. This is because performing clinical trials in children has been associated with ethical and practical issues, which leads to a dearth of safety data during the time of market approval that leads to increased dependence on post-marketing surveillance [36]. Likewise, older patients with polypharmacy, comorbidities, and altered pharmacokinetics are a group with a high risk of ADRs but vulnerable due to underrepresentation in clinical trials and possible unusual manifestations of the adverse events. Resource limitations are one of the inherent issues of pharmacovigilance in most of the environments, especially LMICs. There is lack of enough funding, limited number of people who are trained and adequate infrastructure to in place to promote development of effective reporting system in these countries. Some of these challenges have been dealt with by the WHO efforts to create regional pharmacovigilance centers and offer technical support, but major differences in the pharmacovigilance capabilities remain across the world [19]. Increased difficulties of ADR monitoring have been generated by the globalization of the pharmaceutical markets. Approved medications in one nation can become quickly used in other nations, which puts a strain on regulatory systems to observe safety in a variety of populations and healthcare environments. Fraudulent and substandard medicines also complicate the process of pharmacovigilance in most countries and this necessitates expert methods to detect and act.

## Special populations in pharmacovigilance:

The special populations such as pediatric patients, geriatric patients and pregnant patients have unique traits and weaknesses that need to be dealt with using the pharmacovigilance methods. These populations tend to have other negative adverse drug reaction patterns, need special monitoring plans, and pose unique difficulties concerning drug safety evaluation. The pharmacovigilance of children is especially a challenge owing to the developmental variations in the absorption, distribution, metabolism, and excretion of various drugs among children in different ages. Children are not just small adults their systems are not developed in the same way and this poses age-related susceptibility to adverse drug reactions [37]. The legal and moral restrictions of the clinical tests, which are conducted on children, lead to the fact that a large number of drugs are used off-label in children, and there is insufficient data on their safety when they are actually approved on the market. This gap in knowledge makes post-marketing surveillance more relied on to identify ADRs which are specific to children. In a number of countries, special pediatric pharmacovigilance centers have been organized to cope with these issues. The Pediatric investigation plans of the European Medicines Agency mandate methodical evaluation of children medicines, but this has not been completely extended to all member countries [38]. In the United States, the Pediatric Research Equity Act requires that drugs with a new structure undergo pediatric trials to enhance but not erase the knowledge gaps regarding how the drug is safe in children. Although these regulatory efforts have been made, the under-reporting of pediatric ADRs is still a major issue, and research findings indicate that the rate of reporting may be even less than in adults. Another vulnerable population with special pharmacovigilance requirements is geriatric patients. New physiological changes, the presence of various comorbid conditions, and polypharmacy increase the risk profile of adverse drug reactions in older adults [43]. Inappropriate prescribing of elderly patients is also extremely high, which only adds to the susceptibility of elderly individuals to medication-related injuries. This population may have cognitive impairment and communication challenges that create complications in the recognition and reporting of ADRs. Medication safety programs among elderly patients have been introduced by a number of countries. The Beers Criteria in the United States and the STOPP/START criteria in Europe offer the advice on potentially inappropriate medications in older adults, but have had mixed effects on the actual prescribing practice and ADR rates [40]. Clinical pharmacist-led comprehensive medication review programs have been shown to be effective in reducing inappropriate prescribing and preventing ADRs in older adults, but the use of these programs has not been extensive due to resource constraints. Pregnant women constitute a very difficult population in regard to pharmacovigilance because of the ethical considerations which restrict the use of clinical trials and the possibility of teratogenic effect. The tragedy of thalidomide served to emphasize the disastrous independent of poor drug safety tracking in pregnancy, but a lot remains unknown about the safety of the majority of drugs at pregnancy [41]. Specific maternal-fetal medicine pregnancy registries as well as pharmacovigilance have been developed in a number of countries, but tend to encounter difficulties with enrollment, follow-up, and confounding. The growing sophistication of contemporary pharmacotherapy poses further problems to special populations. Such vulnerable groups could have distinct safety profiles of biologic medicines, gene therapies, and personalized medicine approaches. The incorporation of pharmacogenomic-related information in pharmacovigilance is a nascent strategy of determining genetic risk factors of ADRs among special populations [42]. This is a promising approach to drug safety involving precision medicine that costs a significant amount of money in terms of infrastructure and expertise. Another special population with special pharmacovigilance needs is patients with rare diseases. Orphan drugs are frequently not well-informed about their safety since they are developed on small populations and expedited regulatory programs. Post-marketing surveillance is of especially high importance to these drugs but it is difficult to detect any signals because of the limited number of exposed patients [43]. The pharmacovigilance of rare diseases must involve international cooperation and patient registries.

## Discussion:

The world situation with ADR reporting and monitoring demonstrates a considerable improvement since the formal pharmacovigilance systems were established in the second part of the 20th century. The PIDM of the WHO has been able to establish a framework of collective international drug safety surveillance, VigiBase is a valuable tool used to identify signals and evaluate them [9]. Efforts of harmonization of pharmacovigilance practices especially in Europe have shown to be beneficial of standardized practices to pharma-vigilance though national centers are the primary centers of contact [14]. These advancements have established a more resilient world-wide system of drug safety victimization than in the past. Although these improvements have happened, in our review we can continuously identify the challenges that are still affecting the power of the existing ADR monitoring systems. Underreporting is a worldwide issue and it has been estimated that only 5-10 percent of all ADRs are recorded through spontaneous reporting system [4]. The occurrence imposes severe blind spots in drug safety evaluation and could postpone the safety signal identification. The issue seems to be especially urgent in LMICs, where resource shortage, poor healthcare infrastructure, and other health priorities hinder the establishment of the efficient pharmacovigilance systems [19]. It establishes an unequal system of drug safety surveillance across the globe, and the patient outcomes all over the world may be affected by these disparities. The fact that pharmacovigilance is being integrated with digital technologies is a promising trend that can help in solving certain long-standing issues. New opportunities are provided by the electronic health record, mobile app, and artificial intelligence methods to identify and analyze ADR [26, 27]. Sentinel System by the FDA illustrates the possible potential of active surveillance with the help of large healthcare databases to supplement traditional spontaneous reporting [16]. Yet, our review insinuates that these technological solutions are not panaceas; they introduce other issues of the quality of data, algorithmic bias, and the digital divide, which can further enhance the existing differences in the pharmacovigilance capacity. ADR monitoring still faces certain challenges with special populations. Children, older patients, and pregnant women usually exhibited varied adverse drug reactions patterns compared to general population, and they are still underrepresented in clinical trials and may have unusual manifestations of adverse events [37, 39, 41]. The rising sophistication in contemporary pharmacotherapy such as the use of biologic drugs and combination therapy, further complicates the role of safety evaluation in these susceptible groups. We have found in our review that specialized methods of pharmacovigilance in special populations are required, such as special reporting, active surveillance, and the use of pharmacogenomic information.

Pharmaceutical markets have become globalized, which has posed opportunities as well as challenges of ADR monitoring. On the one hand, the cross-border exchange of safety information can be achieved with the help of the fast dissemination of safety information across the borders by the international cooperation through the WHO PIDM and other networks [12]. Conversely, the speed in which new drugs are being distributed in different healthcare settings is putting strain on regulatory systems that have different capabilities. Imitated and sub-standard drugs also increase the problems of pharmacovigilance in most areas and necessitate special methods of detection and action. A number of gaps in literature are worth being considered in future studies. On the one hand, more systematic assessment of the various pharmacovigilance strategies in various healthcare contexts is necessary to determine the most effective practices and setting variables that affect the effectiveness. Second, studies are required to optimize the incorporation of digital technologies into the pharmacovigilance system and overcome the issues of the quality of data, data security, and fair access. Third, further efforts are required to enhance special population pharmacovigilance methods, especially at resource constrained locations. Lastly, studies on the development of strategies to enhance pharmacovigilance in LMICs, with possible approaches of innovative collaboration and technology transfer, are required. Our review has significant clinical implications. It is also advisable that healthcare professionals should recognize the weakness of the existing monitoring systems of ADR and the implications of providing contributions to pharmacovigilance by means of systematic reporting. Clinicians who draw special populations must be more alert to the possible adverse events that are not reflected by the current monitoring mechanisms. The inclusion of pharmacovigilance data in clinical decision support systems is one of such opportunities that could turn the safety information into the care-point practice. As a research point, our review demonstrates that methodological novelty in ADR detection and assessment is necessary. Scientific foundation of pharmacovigilance would be strengthened through the creation of more advanced ways of assessing causality, detecting of signals, and quantifying risks. The combination of the various data sources, such as the genomic data, the patient-reported outcomes, and the real-life evidence, is one of the promising trends in the future research.

## Conclusion:

The current state of Global ADR reporting and monitoring systems has been greatly advanced since their introduction, and still a great deal of challenges is encountered in realizing all-inclusive drug safety surveillance. The incorporation of digital technologies and specific strategies of vulnerable population suggests some hopeful perspectives of the future development. The persistent differences in effectiveness of pharmacovigilance among various regions and medically specific situations will have to be addressed through international collaboration and capacity building.
